# Sociodemographic and Clinical Factors Associated with Tuberculosis Mortality in Hamadan Province, Iran

**DOI:** 10.34172/jrhs.9193

**Published:** 2025-09-15

**Authors:** Salman Khazaei, Sedigheh Mafakheri, Sanaz Omidi, Abouzar Raeisvandi, Aida Zahiri, Shaghaiegh Zahiri, Fatemeh Torkaman Asadi

**Affiliations:** ^1^Department of Epidemiology, School of Public Health, Hamadan University of Medical Sciences, Hamadan, Iran; ^2^Research Center for Health Sciences, Hamadan University of Medical Sciences, Hamadan, Iran; ^3^Student Research Committee, Hamadan University of Medical Sciences, Hamadan, Iran; ^4^Infectious Disease Research Center, Hamadan University of Medical Sciences, Hamadan, Iran

**Keywords:** Tuberculosis, Mortality, Sociodemographic factors, Clinical predictors, Iran

## Abstract

**Background:**

Tuberculosis (TB) remains a global health concern with high mortality despite treatment options. Understanding the underlying risk factors for TB mortality is essential for guiding effective control strategies. This study examined sociodemographic and clinical factors related to TB mortality in Hamadan province, Iran, to inform control strategies.

**Study Design:**

A cross-sectional study.

**Methods:**

This study evaluated data (March 2011–March 2022) obtained from a provincial TB surveillance database, encompassing smear-positive pulmonary TB (SPT), smear-negative pulmonary TB (SNT), and extrapulmonary TB (EPT) patients. Demographic and clinical characteristics were investigated, and the death rate for each group was calculated by dividing the number of TB-related deaths by the total number of diagnosed TB cases for that group during the study period. Logistic regression was applied to computed unadjusted and adjusted odds ratios (ORs) with a 95% confidence interval for the death rate using Stata 17 (*P*<0.05).

**Results:**

Among the 942 patients included in the study, 49%, 21%, and 30% were diagnosed with SPT, SNT, and EPT, respectively. The risk of mortality was the highest among SPT patients, with EPT cases showing significantly lower odds of death (OR: 0.38, *P*<0.001) compared to SPT. Among SPT patients, mortality was associated with older age (OR: 1.04, *P*<0.001) and positive sputum smear at month 2 (OR: 19.72, *P*<0.001). Human immunodeficiency virus (HIV) positivity significantly increased the death rate in SNT patients (*P*=0.037). In EPT patients, mortality was linked to male gender (*P*=0.042), referral unit (*P*=0.023), TB hospitalization (*P*=0.018), and advanced age (*P*<0.001).

**Conclusion:**

Targeted interventions focusing on early diagnosis, HIV management, and care for high-risk groups (e.g., elderly) are essential to reduce TB mortality in Hamadan province. However, the findings should be interpreted with caution due to limitations, such as reliance on retrospective registry data, potential information bias, and missing data, particularly regarding HIV status.

## Background

 Tuberculosis (TB) remains a significant global health concern.^1^ According to the World Health Organization (WHO), approximately 8.2 million people were newly diagnosed with TB, and 1.25 million deaths occurred in 2023. In this year, TB regained its position as the world’s leading cause of death from a single infectious agent. Despite being curable and preventable, TB remains a significant health challenge across all countries and age groups. In addition, it is a major contributor to deaths related to antimicrobial resistance.^2^

 The global campaign against TB has spurred significant advancements in multifaceted research on the disease. Despite these efforts, we continue to grapple with inadequate diagnostic, therapeutic, and preventive measures. Current projections indicate that existing TB control strategies will fall short of the ambitious 2015-2035 targets, with a 95% reduction in mortality, a 90% decrease in incidence, and a 90% cure rate for patients on first-line treatment. Achieving these goals necessitates a substantial intensification of research and development initiatives. Moreover, it is crucial to enhance the capacity of health systems for early case detection and to elevate the standards of care, diagnosis, and treatment for individuals affected by TB.^3,4^

 Researchers have uncovered crucial predictive factors across various domains that influence TB treatment outcomes, including patient demographics such as age, gender, educational background, and employment status. Additionally, underlying health conditions, particularly diabetes mellitus and human immunodeficiency virus (HIV) infection, play a significant role. The TB disease profile itself also contributes to treatment outcomes, encompassing aspects like sputum and radiological results, the location of the TB infection within the body, and whether the patient has a history of previous TB treatment.^5-9^

 Iran has made notable progress in TB control, with GBD data demonstrating a decline in TB incidence and mortality between 2010 and 2019. However, disease distribution varies regionally. In Hamadan province, western Iran, although the incidence rates of both pulmonary and extrapulmonary TB (EPT) have declined in recent years, our analysis revealed a significant increase in the TB recurrence rate among females, with an average annual percentage change of 18.45% (95% confidence interval [CI]: 3.23–46.47, *P *= 0.001) during 2011–2022, highlighting the need for targeted, gender-specific interventions.^10^

 While existing literature in Iran has predominantly focused on treatment outcomes, there remains a significant lack of evidence on mortality-specific determinants, especially studies leveraging detailed clinical and demographic variables from regional TB surveillance data. The absence of such localized evidence hampers the ability of health authorities to design and implement context-specific interventions. Identifying vulnerable population subgroups and modifiable clinical risk factors is, therefore, essential to inform provincial-level policy and enhance TB mortality prevention efforts. In response to this need, the current study investigates the demographic and clinical predictors of TB-related mortality in Hamadan province using comprehensive registry-based data spanning over ten years. Understanding factors associated with TB mortality is crucial for improving patient outcomes and developing more effective prevention and treatment strategies.

## Methods

 This cross-sectional study was conducted in Hamadan province, located in the west of Iran. It evaluated the association between demographic and clinical characteristics and TB-related mortality across different TB types, including smear-positive pulmonary TB (SPT), smear-negative pulmonary TB (SNT), and EPT. The data were collected from the provincial TB surveillance database, including records obtained from all registered TB patients from March 2011 to March 2022.

 The study included all patients diagnosed with TB during the specified period. Patients were classified into three groups based on their TB types (SPT, SNT, and EPT). The exclusion criteria included non-native individuals, imported cases, and patients with misdiagnosis or diagnostic errors.

 According to Iran’s national TB control guidelines, the diagnosis of SPT is based on the detection of acid-fast bacilli in sputum smears. SNT is diagnosed based on clinical symptoms, radiographic findings, and response to treatment in the absence of smear positivity. EPT is diagnosed through clinical evaluation, imaging, and laboratory confirmation (histopathology, cytology, or microbiology) of specimens from affected sites. TB-related deaths were defined as those occurring during anti-TB treatment unless clearly attributed to other causes and were recorded in the registry system using clinical records and death certificates, consistent with national and the WHO definitions.

 Demographic and clinical information was collected for each patient, including age, gender, location (urban or rural), HIV status based on diagnostic tests, referral unit, treatment group (new or relapse cases), and hospitalization status. The data were extracted from medical records and the provincial TB registry. Regarding missing data, only HIV status had a substantial proportion of unknown values (48.5%). All other variables, including age, gender, TB types, referral unit, treatment group, and hospitalization status, had less than 1% missing data. Due to this minimal level of missingness, no imputation was performed, and the dataset was considered complete for statistical modeling.

 The death rate for each group was calculated by dividing the number of TB-related deaths by the total number of diagnosed TB cases for that group during the study period. Chi-square tests were employed to assess associations between categorical variables and death rates. Logistic regression was used to estimate unadjusted and adjusted odds ratios (ORs) with 95% CIs and identify factors associated with mortality. A *P*-value of less than 0.05 was considered statistically significant. The data were analyzed using Stata software, version 17.

## Results

Table 1 presents the distribution of various demographic and clinical factors among patients with SPT, SNT, and EPT. Based on the results, gender distribution was relatively similar across the three types, with a slight male predominance in SPT and SNT. Most patients were from urban areas across all types. HIV status was unknown for a large proportion of patients, and among those with known status, HIV positivity was low. The network system (subordinate hospital units) was the most common referral unit for all types. The majority of cases across all types were new cases rather than relapses, with EPT having the highest proportion of new cases (98.59%). Age distribution varied significantly among the types, with EPT having a higher proportion of younger patients compared to SPT and SNT (*P *< 0.001).

**Table 1:** Distribution of variables among different types of tuberculosis

**Variables**	**SPT**		**SNT**		**EPT**		***P*** **-value**	**Total**	
	**n**	**%**	**n**	**%**	**n**	**%**		**N**	**%**
Gender							0.075		
Male	251	54.33	111	56.63	134	47.18		496	52.65
Female	211	45.67	85	43.37	150	52.82		446	47.35
Location							0.130		
Urban	288	62.34	118	60.20	194	68.31		600	63.69
Rural	174	37.66	78	39.80	90	31.69		342	36.31
HIV status							0.270		
Yes	18	3.90	7	3.57	6	2.11		31	3.29
No	225	48.70	102	52.04	127	44.72		454	48.20
Unknown	219	47.40	87	44.39	151	53.17		457	48.51
Referral unit							0.001		
Network system (subsidiary outpatient units)	117	25.32	42	21.43	66	23.24		225	23.89
Private clinic	61	13.20	39	19.90	79	27.82		179	19.00
Social welfare	9	1.95	1	0.51	2	0.70		12	1.27
Network system (subordinate hospital units)	223	48.27	100	51.02	122	42.96		445	47.24
Self-reported	23	4.98	4	2.04	3	1.06		30	3.18
Prison	9	1.95	2	1.02	1	0.35		12	1.27
Other	20	4.33	8	4.08	11	3.87		39	4.14
TB hospitalization							0.150		
Yes	193	41.77	69	35.20	102	35.92		364	38.64
No	269	58.23	127	64.80	182	64.08		578	61.36
Treatment group							0.001		
New case	417	90.26	183	93.37	280	98.59		880	93.42
Relapse case	45	9.74	13	6.63	4	1.41		62	6.58
Age group (year)							0.001		
≤ 18	6	1.30	8	4.08	18	6.34		32	3.40
19-64	207	44.81	87	44.39	207	72.89		501	53.18
65 +	249	53.90	101	51.53	59	20.77		409	43.42

*Note*. TB: Tuberculosis; HIV: Human immunodeficiency virus; SPT: Smear-positive pulmonary; SNT: Smear-negative pulmonary; EPT: Extrapulmonary TB.

 A total of 131 TB-related deaths occurred, including 84, 25, and 22 in SPT, SNT, and EPT patients, respectively. Based on the obtained data (Table 2), statistically significant associations were found between mortality and referral unit, TB hospitalization, and age group among SPT patients (*P *< 0.001). The highest death rates were observed among patients referred from subordinate hospital units (27%), those hospitalized for TB (29%), and those aged 65 and older (26%). No significant associations were found with gender, HIV status, or treatment group (*P *> 0.05). In SNT patients, a statistically significant association was observed between mortality and HIV status (*P *= 0.037), with the highest death rate belonging to HIV-positive individuals (43%). Other variables, including gender, place of residence, referral unit, TB hospitalization, treatment group, and age group, showed no significant associations with mortality (*P* > 0.05). Among EPT patients, mortality was significantly associated with gender (*P *= 0.042), referral unit (*P *= 0.023), TB hospitalization (*P *= 0.018), and age group (*P *< 0.001). No significant associations were found with place of residence or HIV status (*P *> 0.05).

**Table 2:** Association between mortality and risk factors in SPT, SNT, and EPT patients

**Variables**	**Death in SPT patients**			**Death in SNT patients**			**Death in EPT patients**		
	**n**	**%**	***P *** **value**	**n**	**%**	***P *** **value**	**n**	**%**	***P *** **value**
Gender			0.125			0.433			0.042
Male	52	0.21		16	0.14		15	0.11	
Female	32	0.15		9	0.11		7	0.05	
Location			0.930			0.076			0.333
Urban	52	0.18		11	0.09		13	0.07	
Rural	32	0.18		14	0.18		9	0.10	
HIV status			0.544			0.037			0.367
Yes	5	0.28		3	0.43		1	0.17	
No	41	0.18		10	0.09		7	0.06	
Unknown	38	0.17		12	0.14		14	0.09	
Referral unit			0.001			0.067			0.026
Network system (subsidiary outpatient units)	14	0.12		-			2	0.30	
Private clinic	3	0.05		5	0.13		2	0.03	
Social welfare	-			-			-		
Network system (subordinate hospital units)	61	0.27		19	0.19		18	0.15	
Self-reported	4	0.17		1	0.25		-		
Prison	2	0.22		-			-		
Other	-			-			-		
TB hospitalization			0.001			0.328			0.018
Yes	56	0.29		11	0.16		13	0.13	
No	28	0.10		14	0.11		9	0.05	
Treatment group			0.206			0.571			0.190
New case	79	0.19		24	0.13		21	0.08	
Relapse case	5	0.11		1	0.08		1	0.25	
Age group (year)			0.001			0.530			0.001
≤ 18	-			-			1	0.06	
19-64	20	0.09		11	0.12		7	0.03	
65 +	64	0.26		14	0.14		14	0.23	

*Note*. TB: Tuberculosis; HIV: Human immunodeficiency virus; SPT: Smear-positive pulmonary TB; SNT: Smear-negative pulmonary TB; EPT: Extrapulmonary TB.

 There was a significant association between TB type and mortality (Table 3). Compared to SPT, EPT was associated with a significantly lower death rate (OR: 0.38, 95% CI: 0.23–0.62, *P *< 0.001). In other words, the odds of mortality in patients with EPT were 62.3% lower than those in patients with SPT. SNT also demonstrated a lower death rate compared to SPT, though this difference was not statistically significant (OR: 0.66, 95% CI: 0.41–1.06, *P *= 0.088), implying that the odds of mortality in SNT patients were 34.3% lower than in SPT patients.

**Table 3:** Predictors of mortality in SPT patients using unadjusted and adjusted odds ratio for other variables in the model

**Variables**	**Unadjusted OR (95% CI)**	***P*** ** value**	**Adjusted OR (95% CI)**	***P*** ** value**
Age (year)	1.04 (1.02–1.05)	0.001	1.04 (1.02–1.07)	0.001
Gender				
Male	Ref.	0.009	Ref.	0.240
Female	0.60 (0.41–0.88)		0.61 (0.26–1.39)	
Location				
Urban	Ref.	0.146		
Rural	1.32 (0.91–1.92)			
HIV status				
Yes	Ref.		Ref.	
No	0.36 (0.16–0.82)	0.014	0.23 (0.03–1.77)	0.159
Unknown	0.39 (0.18–0.90)	0.028	0.18 (0.02–1.46)	0.109
Referral unit				
Subsidiary outpatient units	Ref.			
Private clinic	0.77 (0.34–1.75)	0.536		
Subordinate hospital units	3.69 (2.12 –6.43)	0.001		
Self-reported	2.61 (0.88–7.74)	0.083		
Prison	2.61 (0.53–12.95)	0.240		
TB hospitalization				
Yes	Ref.		Ref.	
No	0.34 (0.24–0.50)	0.001	0.41 (0.14–1.18)	0.098
Chest X-ray results				
Non-suggestive	Ref.			
More suggestive	1.74 (0.92–3.29)	0.091		
Hospitalization time (day)	1.04 (1.02–1.06)	0.001	1.02 (0.98–1.07)	0.299
SSC month 2				
Negative	Ref.			
+ 1	6.41 (3.56–11.54)	0.001	Ref.	
Basil 1-9	0.46 (0.11 –1.99)	0.297	19.72 (7.85–49.57)	0.001
SSC before treatment				
+ 1	Ref.		1.07 (0.20–5.62)	0.934
+ 3	0.86 (0.463–1.59)	0.631		
Basil 1-9	2.01 (0.94–4.28)	0.070		
+ 2	1.26 (0.62–2.55)	0.522		
Time from sign initiation to diagnosis (day)	1.041 (1.02–1.06)	0.001		

*Note*. TB: Tuberculosis; HIV: Human immunodeficiency virus; SPT: Smear-positive pulmonary TB; SNT: Smear-negative pulmonary TB; EPT: Extrapulmonary TB; SSC: Sputum smear concentration; OR: Odds ratio; CI: Confidence interval.

Table 3 lists unadjusted and adjusted ORs for predictors of mortality in patients with SPT, respectively. In the unadjusted analysis, significant predictors of mortality included older age, male gender, HIV status, referral from subordinate hospital units, TB hospitalization, longer hospitalization time, and positive sputum smear at month 2 (*P *< 0.05). After adjustment, age remained a significant predictor (OR: 1.04, 95% CI: 1.02–1.07, *P *= 0.001), as did positive sputum smear at month 2 (OR: 19.72, 95% CI: 7.85–49.57, *P *< 0.001). Other factors, such as gender, location, HIV status, and TB hospitalization, represented no statistical significance in the adjusted model, indicating potential confounding effects (*P *> 0.05).

## Discussion

 This study investigated the sociodemographic and clinical factors associated with TB mortality in Hamadan province, Iran. In the present study, a significant association was observed between mortality in SNT patients and HIV infection. A combination of factors likely explains the higher TB death rate in HIV-infected individuals, including advanced immunosuppression,^11^ rapid disease progression due to ineffective immune responses in controlling mycobacterium TB growth, and delayed diagnosis and treatment of TB infection due to low disease prevalence and common negative sputum results.^12^ The other factors are delayed HIV infection identification due to social stigma or inadequate acceptance of HIV testing in TB clinics,^12^ delayed treatment initiation or lack of access to antiretroviral therapy,^11^ and high rates of multidrug-resistant TB preventing timely effective treatments.^13^

 The results of a study in the United States revealed that SNT in HIV patients was not associated with higher mortality.^14^ This is because SNT can be routinely and definitively diagnosed in the US, while high-burden countries often rely solely on acid-fast bacilli microscopy, potentially leading to delayed diagnosis, treatment, and higher mortality.^14^ Therefore, health managers and planners need to develop and implement specific guidelines and policies for early HIV and TB diagnosis and initiation of antiretroviral treatments.^11^ HIV status was not associated with mortality in EPT patients in our study, contrary to some studies that identified HIV as an independent risk factor for death in EPT patients.^15,16^ The small sample size of HIV-positive EPT patients (n = 6) likely limited statistical power, increasing the probability of a Type II error and limiting firm conclusions.

 Studies on gender and its relationship with mortality in EPT patients have shown conflicting results. In our study, male gender was a risk factor. A study in Texas found female gender to be a risk factor for mortality in EPT TB patients,^15^ while another study in Ghana reported no association between gender and mortality in EPT patients.^16^ In our study, gender was not related to mortality in SNT and SPT patients. Some studies indicated that there was no association between gender and mortality in SPT patients,^17^ while others identified gender as a risk factor for death in pulmonary TB patients.^18^ Possible explanations for higher mortality among male EPT patients may include differences in health-seeking behavior, delayed diagnosis, and higher prevalence of comorbidities or risk factors such as smoking, alcohol use, and occupational exposures among men.

 In this study, age was a significant predictor of mortality in SPT patients, with older patients at higher risk. Studies have shown that mortality in SPT patients increases with age ^17^. In older individuals, comorbidities such as diabetes, malignancies, cardiovascular disorders, history of gastrectomy, and other diseases may alter TB symptoms.^19^ Unusual radiological findings in older adults, compared to younger individuals, can delay diagnosis and subsequent treatment.^20^ Atypical clinical features in older adults may be confused with age-related diseases, and treatment can be associated with drug side effects.^21^ Older age is a factor for persistent smear positivity in SPT patients, increasing the risk of adverse outcomes.^22^ Higher mortality in older SPT patients may be due to poor living conditions, including poverty and malnutrition. Older adults are more likely to suffer from malnutrition.^23^

 Our findings confirmed that a positive sputum smear at month 2 of treatment was a significant predictor of death rate in SPT patients. In SPT patients, sputum smear conversion is a key indicator of treatment response and infection reduction.^24^ Positive smear and culture after treatment prolong infection and are associated with adverse outcomes,^25^ predicting relapse and treatment failure.^26^ Risk factors for positive sputum smear at month 2 of treatment include male gender, low body mass index, HIV infection, disease duration over 2 months, cavity or extensive disease on chest radiography, higher smear grade, and higher colony counts in culture.^25,26^

 The results of this study showed that EPT patients had a lower death rate compared to SPT patients, which conforms to the results of another research.^27^ Even after successful treatment, SP TB has adverse effects on patient survival and reduces it.^28^ A study in South Africa found a significantly higher death rate in EPT patients compared to pulmonary TB patients, in smear-negative compared to smear-positive cases, and in retreatment cases.^29^

 This study offers several strengths and valuable applications. It provided a comprehensive analysis of TB mortality factors across different TB types in Hamadan province, Iran, using data from 2011 to 2022. The large sample size and extended timeframe enhanced the reliability of the findings. By identifying specific risk factors for each TB type, the study offers crucial insights for targeted interventions and personalized care strategies. These findings can directly inform local and national TB control programs, helping prioritize resources and improve patient outcomes.

 Nonetheless, this study had several limitations that should be considered when interpreting its results. First, as a retrospective analysis of registry data, it may be subject to information bias and missing data, particularly evident in the high proportion of unknown HIV status among patients. Additionally, the reliance on routinely collected data implies that some potentially important factors influencing TB mortality, such as socioeconomic status, education level, or comorbidities other than HIV, were not available for analysis. Although certain factors (e.g., age, sputum smear status, and HIV infection) were statistically significant, their independent predictive values can be overestimated in the absence of adjustment for underlying diseases. Finally, the cross-sectional nature of the study precludes drawing causal inferences about the relationships observed between various factors and TB mortality.

**Highlights**

- Age and sputum positivity at month 2 were strong independent predictors of mortality in SPT cases.
- HIV positivity significantly increased the death rate in SNT patients.
- In EPT patients, mortality was linked to male gender, referral unit, TB hospitalization, and advanced age.
- EPT patients had significantly lower odds of mortality compared to SPT patients.

## Conclusion

 For SPT, age and positive sputum smear at month 2 of treatment were significant predictors of mortality. HIV status emerged as a crucial factor for SNT mortality. In EPT cases, gender, referral unit, TB hospitalization, and age group significantly influenced death rates. Notably, EPT patients demonstrated a lower death rate compared to SPT patients. These results underscore the need for targeted interventions and personalized care strategies, particularly for high-risk groups, such as older patients, those with persistent positive sputum smears, and HIV-positive individuals.

 The study also highlights the importance of early detection, proper referral systems, and comprehensive HIV testing and management in TB care. For the healthcare system in Hamadan province and similar regions in Iran, it is recommended that several measures be taken to manage severe or complicated TB cases. They include implementing systematic early detection programs for high-risk patients, ensuring routine follow-up sputum smears to monitor treatment response, integrating TB and HIV services with universal HIV screening in TB patients, and reinforcing the capacity of referral units, especially subordinate hospital networks. These interventions could significantly improve patient outcomes and reduce TB-related mortality in the region.

## Acknowledgements

 This study was approved and sponsored by the Deputy of Research and Technology of Hamadan University of Medical Sciences (Research ID: 1402121510968). We are particularly thankful to the staff of the TB Control Program in Hamadan province for their assistance in collecting data and providing access to the TB register database.

## Competing Interests

 The authors declare that there is no conflict of interests in this study.

## Ethical Approval

 This study was approved by the Ethics Committee of Hamadan University of Medical Sciences (IR.UMSHA.REC.1402.735).

## Funding

 This study was financially supported by Hamadan University of Medical Science, Hamadan, Iran.
